# Variety-Specific Flowering of Sugarcane Induced by the Smut Fungus *Sporisorium scitamineum*

**DOI:** 10.3390/plants12020316

**Published:** 2023-01-09

**Authors:** Liang Shuai, Hairong Huang, Lingyan Liao, Zhenhua Duan, Xiaoqiu Zhang, Zeping Wang, Jingchao Lei, Weihua Huang, Xiaohang Chen, Dongmei Huang, Qiufang Li, Xiupeng Song, Meixin Yan

**Affiliations:** 1 College of Food and Biological Engineering, Institute of Food Research, Hezhou University, Hezhou 542899, China; 2 Sugarcane Research Institute, Guangxi Academy of Agricultural Sciences, Sugarcane Research Center, Chinese Academy of Agricultural Sciences, Key Laboratory of Sugarcane Biotechnology and Genetic Improvement (Guangxi), Ministry of Agriculture, Guangxi Key Laboratory of Sugarcane Genetic Improvement, Nanning 530007, China; 3 Biotechnology Research Institute, Guangxi Academy of Agricultural Sciences, Nanning 530007, China; 4 Baise Agricultural Scientific Research Institute, Baise 533612, China

**Keywords:** flower, smut disease, identification, *S. scitamineum*, flowering-related genes, sugarcane

## Abstract

Sugarcane smut is the most severe sugarcane disease in China. The typical symptom is the emerging of a long, black whip from the top of the plant cane. However, in 2018, for the first time we observed the floral structures of sugarcane infected by smut fungus in the planting fields of China. Such smut-associated inflorescence in sugarcane was generally curved and short, with small black whips emerging from glumes of a single floret on the cane stalk. Compatible haploid strains, named Ssf1-7 (*MAT-1*) and Ssf1-8 (*MAT-2*), isolated from teliospores that formed black whips in inflorescence of sugarcane were selected for sexual mating assay, ITS DNA sequencing analysis and pathogenicity assessment. The isolates Ssf1-7 and Ssf1-8 showed stronger sexual mating capability than the reported *Sporisorium scitamineum* strains Ss17 and Ss18. The ITS DNA sequence of the isolates Ssf1-7 and Ssf1-8 reached 100% similarity to the isolates of *S. scitamineum* strains available in GenBank. Inoculating Ssf1-7 + Ssf1-8 to six sugarcane varieties, i.e., GT42, GT44, GT49, GT55, LC05-136 and ROC22, resulted in different smut morphological modifications. The symptoms of floral structure only occurred in LC05-136, indicating that the flowering induction by *S. scitamineum* is variety-specific. Furthermore, six selected flowering-related genes were found to be differentially expressed in infected Ssf1-7 + Ssf1-8 LC05-13 plantlets compared to uninfected ones. It is concluded that the flowering induction by *S. scitamineum* depends on specific fungal race and sugarcane variety, suggesting a specific pathogen–host interaction and expression of some flowering-related genes.

## 1. Introduction

Sugarcane belongs to the C_4_ crop, a perennial tropical and subtropical crop. However, sugarcane smut, one of the most important diseases worldwide, causes enormous damage to sugarcane during its active growing stage [1]. Notably, sugarcane smut is the most severe sugarcane disease in China [2,3,4], which seriously reduces the cane yield and sugar content, and results in great economic losses to the sugarcane industry in China [5,6]. The disease was first reported in Natal, South Africa, in 1877 [7], and gradually spread in sugarcane-producing countries globally. The most apparent symptoms of sugarcane smut are the emergence of a long, black whip from the top of the cane stalk. The whip structure comprises the plant tissue in the center, surrounded by black teliospores of the smut fungus [8,9]. The causal agent of this disease has been identified as *Sporisorium scitamineum*, which has a similar life cycle to other smut fungi. Teliospores germinate to produce haploid sporidia, and compatible sporidia (*MAT-1*/*MAT-2*) fuse to form pathogenic dikaryotic hyphae to infect plant canes, resulting in the production of diploid teliospores [9,10].

Smut fungus of sugarcane in different populations has been reported to have rich diversity, but the diversity of *S. scitamineum* populations were revealed to be relevant to geographical origin, not to the host [11,12]. In addition, the races of smut fungus *S. scitamineum* have been identified in some sugarcane planting areas or countries worldwide [1,13], such as three races in Hawaii [14], two races in Brazil [15], five races in Pakistan [16] and three races in Taiwan [17]. Recently, three pathogenic races of *S. scitamineum* derived from different geographical groups in mainland China developed highly significant differences in pathogenicity [18,19,20].

Sugarcane can be classified as an intermediate day plant, and sugarcane flowers can be induced when day length decreases [21,22]. Floral induction of sugarcane is determined by internal factors (hormones, circadian rhythm, etc.) and external factors, such as photoperiod, temperature, humidity, etc. [23,24,25]. Many flowering-related genes have been reported to be involved in sugarcane flowering induction, based on transcriptomic analysis under controlled photoperiodic conditions [22]. Some genes were found to be significant differential expression during the 24 h cycle [21]. In short, these reports confirmed that sugarcane flowering is a complex physiological process.

*S. scitamineum* causes several types of morphological modification in sugarcane, such as tillering plantlets with erect shoots, small narrow leaves and bud proliferation [8]. However, in October 2018, we observed a floral infection by smut fungus in sugarcane plants in the field of Sugarcane Research Institute, Guangxi Academy of Agricultural Sciences, Nanning, Guangxi, China. Similar observations in sugarcane have been reported in Australia and India [8,23]. In this study, we further identified that the causal organism of smut fungus *S. Scitamineum* in sugarcane flowering, based on morphological assessment, ITS DNA sequence analysis and pathogenicity assessment. We also found that flowering induction only occurred in a particular combination of sugarcane cultivar and fungal race, accompanied by perception of photoperiod and expression of flowering-related genes, indicating a specific plant–fungal interaction underlying this phenomenon.

## 2. Results

### 2.1. Diseased Symptoms and Morphological Characteristics of Smut Fungus

**Figure 1:**
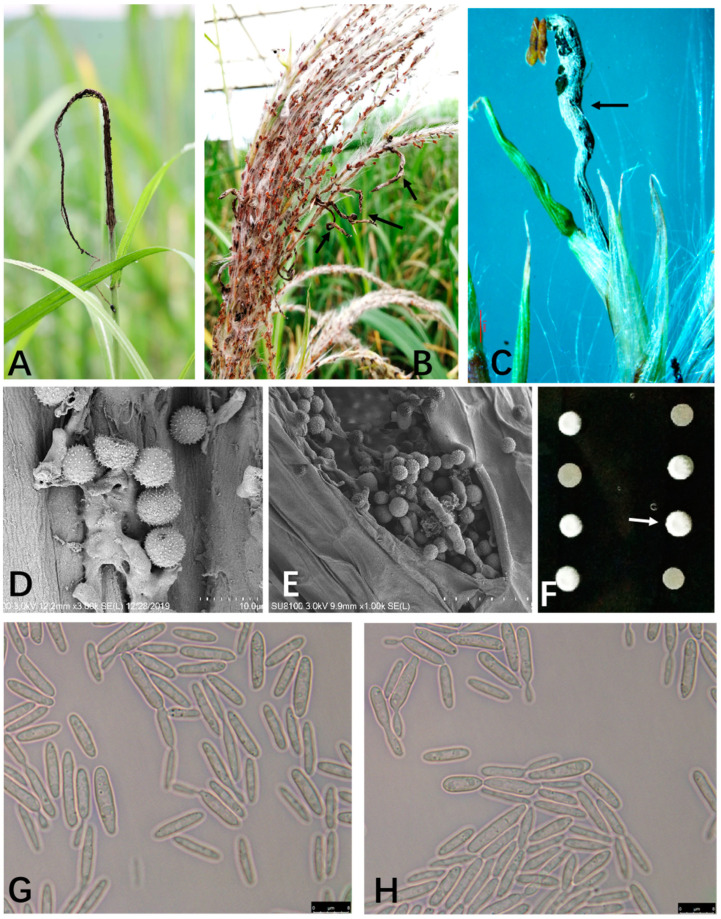
Symptoms of sugarcane smut on flowers and identification of the causal agent. (**A**): Symptom of smut from top of cane; (**B**,**C**): Symptom of inflorescence smut (**arrows**) in sugarcane; (**D**): Scanning electron microscopy of teliospores isolated from the smut from top of stalk (Ss17 and Ss18 infected); (**E**): Scanning electron microscopy of teliospores isolated from the inflorescence smut; F: Mating assay of haploid strains isolated from smut in flower; different mating type haploid strains mixed randomly on YePSA plates, two strains are regard as different mating types (*MAT-1* and *MAT-2*) if they can cross together (“fluffy” colony, arrows), whereas they are the same mating type (both are *MAT-1* or both are *MAT-2*) if they cannot cross (yeast-like colony); (**G**): Microscopic image of haploid sporidia of Ss17; H: Microscopic image of haploid sporidium of Ssf1-7. Scale bar: 10.0 μm in D, 50.0 μm in E, 8.0 μm in G and H.

The common characteristic of sugarcane smut is a black whip-like structure from the top of the stalk (Figure 1A). Sugarcane inflorescence involves bearing several curved, short, and small black whips, emerging from glumes in a single floret (Figure 1B,C). These glume-associated small black whips in the inflorescence ranged from 3–20 mm by measuring from a total of 36 black whips, and the whips were composed of a layer of fungal tissue that developed black teliospores. Under SEM, the teliospores isolated from sugarcane flower smut were spherical with the length of 5.52 to 9.69 µm (*n* = 100) (Figure 1E), which was comparable to the regular teliospores (5.52 to 7.96 µm, *n* = 100) isolated from the diseased cane infected by the established sugarcane smut fungus, such as the Ss17 (*MAT-1*) and Ss18 (*MAT-2*) strains [26] (Figure 1D). The teliospores from the isolates were brown or black, similar to the reported *S. scitamineum* teliospores. Therefore, morphological characteristics of the teliospores isolated from sugarcane flowers were similar to those isolated from the regular cane whips.

On the contrary, haploid sporidia of the opposite mating types were isolated from the teliospores collected from sugarcane flowers. These sporidial strains, i.e., Ssf1-7 and Ssf1-8, were capable of sexual mating and filamentous growth (Figure 1F). Under the microscope, the Ssf1-7 sporidia were cigar-shaped and colorless (Figure 1H), 7.53–18.31 μm in length and 1.73–3.82 μm (*n* = 100) in width, which was comparable to the shape (Figure 1G) and size (9.06 to 20.17 μm ×1.63 to 3.89 μm, *n* = 100) of the reported strain Ss17 sporidia. Strains Ssf1-7 and Ssf1-8 were classified as *MAT-1* and *MAT-2,* respectively, based on the mating assay of crossing with Ss17 (*MAT-1*) and Ss18 (*MAT-2*), respectively. Overall, we obtained the fungal isolates from the sugarcane inflorescence smut and characterized the morphology of the fungal teliospores and sporidia, which were similar to the known sugarcane smut fungus of *S. scitamineum*.

**Figure 2:**
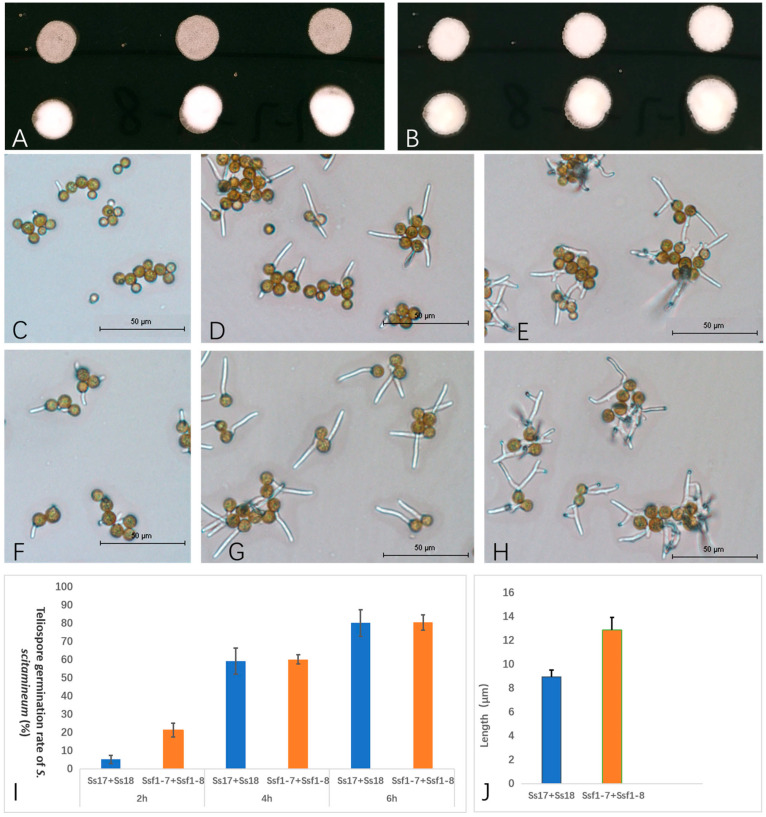
Mating ability and teliospore germination of two different strains of *S. scitamineum*. Mating assay for 24 h (**A**) and 48 h (**B**) respectively. Upper line are mixed cells of Ss17 + Ss18, and lower line are mixed cells of Ssf1-7 +Ssf1-8; (**C**–**E**): Teliospores germination of Ss17 + Ss18 at 2, 4 and 6 h, respectively; (**F**–**H**): Teliospores germination of Ssf1-7 +Ssf1-8 at 2, 4 and 6 h, respectively; (**I**): Teliospore germination rates of different isolates of *S. scitamineum* and (**J**): Length of basidia produced from teliospores of *S. scitamineum* in 4 h. Significant difference (*p* < 0.05) determined by Student’s *t*-test.

To understand whether there is any difference between smut fungus from sugarcane flowers and from the top of cane stalks, we tested the growth of haploid sporidia, mating ability and teliospore germination of both fungal strains. The results showed that the growth rate of haploid cells was comparable (Supplementary Figure S2). However, the newly isolated strains, Ssf1-7 and Ssf1-8, showed more strong sexual mating ability than the reported isolates, Ss17 and Ss18, based on the cross assay. The visible mycelial dikaryotic colonies developed after inoculation of the mixture of Ss17 and Ss18 on YePSA medium at 28 °C for 48 h. In contrast, the mycelial colonies formed from the mixture of Ssf1-7 and Ssf1-8 became visible as early as at 24 h, under the same culture conditions (Figure 2A,B). The germination rate of teliospores formed by the diploid (mating type) strain Ss1-7 + 1-8, derived from Ssf1-7 and Ssf1-8 mating, is higher than that of teliospores created by the diploid (mating type) strain Ss17 + 18, derived from Ss17 and Ss18 mating on 1% agar plates at 28 °C in 2 h (Figure 2C,F,I). The teliospores germination rate of both diploid strains become comparable in 4 h. Still, the length of basidia produced from teliospores formed by the diploid strain Ss17 + 18 is shorter than that of teliospores formed by the diploid strain Ss1-7 + 1-8 (Figure 2D,G,J). This result proved that the newly isolated smut fungus was different from the regular whip smut fungus in mating ability and teliospore germination, especially in sexual mating capability.

### 2.2. Pathogenicity of Smut Fungus Isolated from Sugarcane Inflorescence

**Figure 3:**
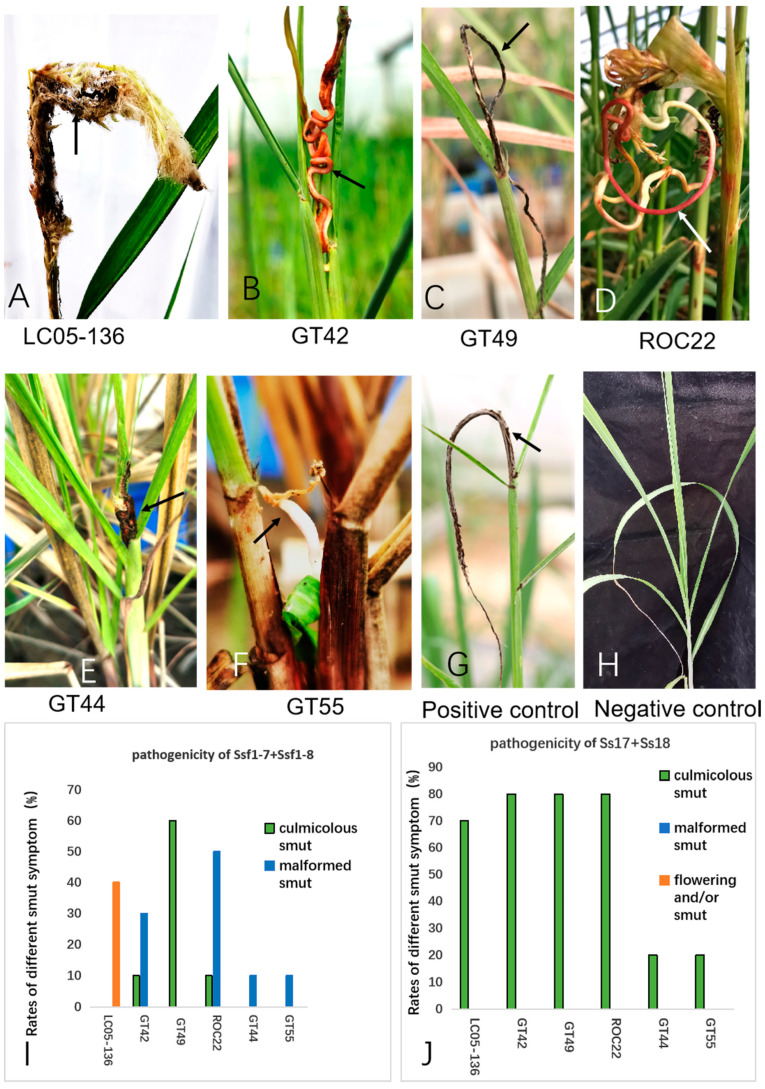
Smut symptoms in different sugarcane varieties. (**A**–**F**): Smut symptoms (arrows) in sugarcane varieties, i.e., LC05-136, GT42, GT49, ROC22, GT44 and GT55, respectively, which was inoculated by fungus cells of the mixture of the isolates of Ssf1-7 and Ssf1-8 from fluorescence smut. (**G**): Characteristic smut symptom (arrows) in sugarcane on the top of stalk inoculated with the mixture of Ss17 and Ss18. (**H**): negative control. (**I**): Smut rates in different sugarcane varieties inoculated smut fungus mixture of Ssf1-7 + Ssf1-8. (**J**): Frequency of smut symptoms development in different sugarcane varieties inoculated smut fungus mixture of Ss17 + Ss18.

To assess the virulence of the Ssf1-7 and Ssf1-8 isolates, we inoculated their 1:1 mixed cells to disease-free tissue-cultured seedlings of six commercial sugarcane varieties, namely GT42, GT44, GT49, GT55, LC05-136 and ROC22. Inoculation of the mixed Ss17 + Ss18 sporidia served as a positive control. The pathogenicity test revealed that the inoculation of the Ssf1-7 and Ssf1-8 mixture resulted in different morphological modifications, with varying disease rates, in the plants of different sugarcane varieties (Figure 3A–I). Inoculation with Ssf1-7 + Ssf1-8 induced flowering, with whip-like structures emerging from the glumes, in 4 out of 10 seedlings for the sugarcane variety LC05-136 4 months post-inoculation (Figure 3A). Regular whips from the top of the plant stalks formed in varieties GT42 (10%), ROC22 (10%) and GT49 (60%) (Figure 3C,I), whereas abnormal morphological modifications developed in the infected varieties of GT42 (30%), ROC22 (50%), GT44 (10%) and GT55 (10%), with less or no teliospores formation (Figure 3D–F,I). The characteristic smut whip structures on the top of plant stalks were induced by inoculation of the mixture of Ss17 and Ss18 in all inoculated sugarcane varieties for four months (Figure 3G,J), and the non-infected plants (negative control) presented vigorous growth (Figure 3H). Teliospores and the derivative sporidia were re-isolated from the inflorescence or top of the black whips, and they were identical to the Ssf1-7 and Ssf1-8 used for inoculation (data not shown). This result confirmed that the smut fungi Ssf1-7 and Ssf1-8 were indeed responsible for inducing sugarcane flowering specifically in the host variety LC05-136; they were also capable of causing chronic sugarcane smut disease symptoms. Therefore, Ssf1-7 and Ssf1-8 belong to *S. scitamineum*.

### 2.3. ITS Identification

**Figure 4:**
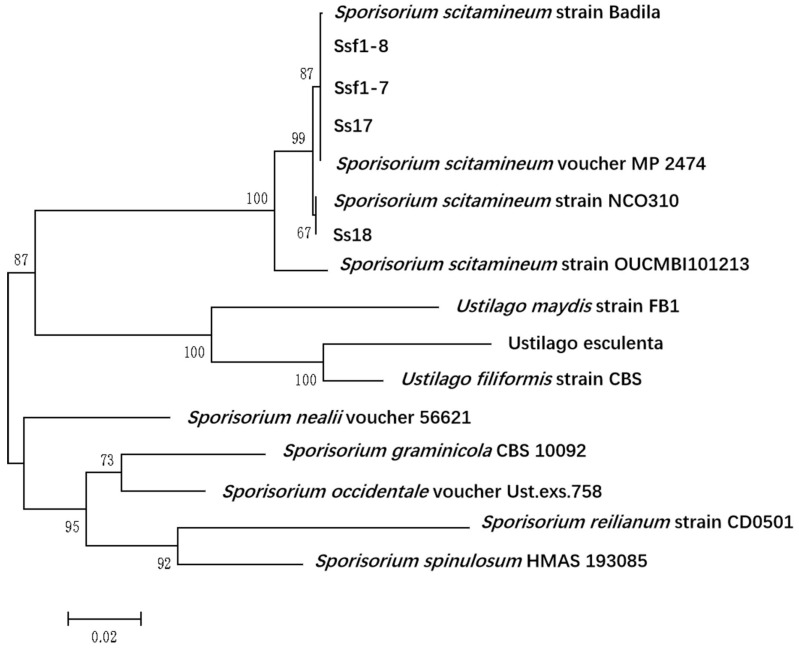
Phylogenetic analysis of ITS DNA sequences of *S. scitamineum* and other smut fungi. The phylogenetic tree is calculated with Maximum Likelihood using Mega ver. 6.0 software based on a Clustal W alignment. The following ITS DNA sequences were chosen for comparison: ITS of Ssf1-7, Ssf1-8, Ss17 and Ss18 of *S. scitamineum*, ITS sequences of *Sporisorium scitamineum* voucher MP 2474, *Sporisorium scitamineum* strain Badila, *Sporisorium scitamineum* strain NCO310, *Sporisorium scitamineum* strain OUCMBI101213, *Sporisorium spinulosum* HMAS 193085, *Sporisorium graminicola* CBS 10092, *Sporisorium nealii* voucher 56621, *Sporisorium occidentale* voucher Ust.exs.758, *Ustilago filiformis* strain CBS, *Sporisorium reilianum* strain CD0501, *Ustilago maydis* strain FB1 and *Ustilago esculenta* (GenBank accession numbers MZ470432, MZ470431, MZ470434, MZ470433, AY345007.1, EF185078.1, EF185069.1, HQ914908.1, NR_119765.1 NR_155843.1, AY740055.1, AY344985.1, MH855347.1, FJ167350.1, KP866233.1 and AB211929.1).

To further confirm whether Ssf1-7 and Ssf1-8 belong to *S. scitamineum*, we performed PCR-amplification of the ITS region of these two strains, using the primer pairs of ITS4/ITS5. The resulting 780bp fragments were sequenced (Supplementary Table S1) and used for BLASTN search in the GenBank database. The amplified ITS sequences reached 100% similarity to the *S. scitamineum* isolates available in GenBank. Phylogenetic analysis (Figure 4) further confirmed that the ITS sequences of Ssf1-7 and Ssf1-8 were most closely related to *S. scitamineum* strains rather than other smut fungi, including *Ustilago maydis, U. hordei* or *S. reilianum*. Based on the morphological characterization, pathogenicity assay and ITS sequence similarity, it is concluded that the causal agent of sugarcane inflorescence smut was *S. scitamineum*.

### 2.4. Flowering Induction by S. scitamineum in Sugarcane Variety LC05-136

**Figure 5:**
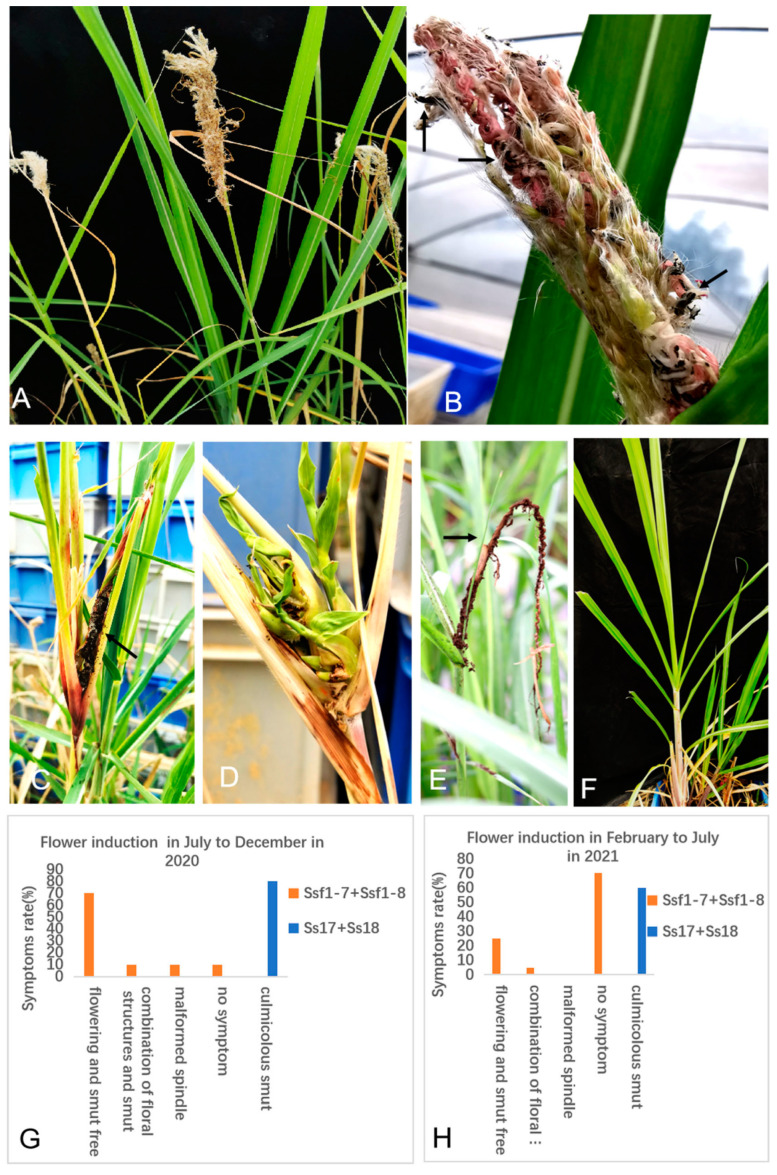
*S. scitamineum* Ssf1-7 and Ssf1-8 induced flowering in sugarcane variety (LC05-136). (**A**): Sugarcane flowering induced by inoculation with the new smut pathogen isolates of Ssf1-7 and Ssf1-8; (B): Symptom of inflorescence smut (arrows) in sugarcane infected by inoculation with the isolates of Ssf1-7 + Ssf1-8; (**C**,**D**): Symptom of malformed spindle in cane plant (arrow show smut); (**E**): Regular symptom of smut (arrow) in sugarcane from the top of canes induced by *S. scitamineum* compatible strains Ss17 and Ss18. (**F**): negative control (water inoculation); (**G**): Flower induction of sugarcane variety inoculated smut fungus mixture of Ssf1-7 + Ssf1-8 in July to December in 2020. H: Flower induction of sugarcane variety inoculated smut fungus mixture of Ssf1-7 + Ssf1-8 in the month of February to July 2021.

To further confirm the flowering of sugarcane variety LC05-136 induced by *S. scitamineum*, sugarcane floral induction assay by inoculation with Ssf1-7 + Ssf1-8 sporidia mixture was performed. Inoculation with the *S. scitamineum* Ss17 + Ss18 mixture was used as a control, and water inoculation as a blank control. The various disease symptoms are shown in Table S2. It was found that 80% of the Ssf1-7 + Ssf1-8 infected plants displayed induced flowering symptoms (Figure 5A) within 6 months, from July to December. Sugarcane seedlings infected by the smut fungus of Ssf1-7 +Ssf1-8 were taller and thinner, with erect shoots and small narrow leaves, compared to non-infected seedlings of an equivalent age. The inflorescence induced by the smut fungus of Ssf1-7 +Ssf1-8 developed on the shoots of plant canes. The first induced flower occurred on the 65th day after inoculation, and most of the rendered flowers occurred within 90 days (Figure 5G, Table S2). Two stalks were induced to produce the symptoms of floral structure and black whips (Figure 5B) from 115 to 136 days, which account for 10% of total infected plants (Figure 5G). Other symptoms, such as malformed spindle, appeared in the 2 plant stalks in 96 and 113 days, and 1 was mixed with smut structure (Figure 5C,D), while the other showed no obvious smut structures. However, 80% of the Ss17 + Ss18-inoculated seedlings developed characteristic smut symptoms of the black whip from the top of the stalks (Figure 5E,G) 120 days post inoculation (Table S2). The water-inoculated plants (negative control) grew vigorously with no smut symptoms (Figure 5F).

On the contrary, the result of the sugarcane floral induction assay performed from February to July showed that 30% of the total tested seedlings of variety LC05-136 induced flowering (Figure 5H), and the induced flower appeared in 110 to 124 days after inoculation with the Ssf1-7 + Ssf1-8 cells mixture (Table S3). One sugarcane stalk was induced to produce the symptoms of the combination of floral structure and black whips. A total of 60% of tested seedlings injected with the mixed Ss17 and Ss18 cells developed characteristic smut symptoms of the black whip from the top of the stalks in 120 days post inoculation (Figure 5H, Table S3). Overall, we confirmed that the identified *S. scitamineum* strains Ssf1-7 and Ssf1-8 were capable of infecting the glumes and inducing flowering to a particular sugarcane variety, LC05-136.

### 2.5. Assessment of Comparative Expression of Flowering Related Genes by RT-qPCR

To further confirm the flowering of sugarcane variety LC05-136 induced by *S. scitamineum* at the molecular level, the expression of 19 flowering-related genes was assessed in infected (including Ssf1-7 + Ssf1-8 and Ss17 + Ss18) and non-infected control seedlings at the beginning of smut symptoms (black whip) and initiating floral structures, using RT-qPCR.

**Figure 6:**
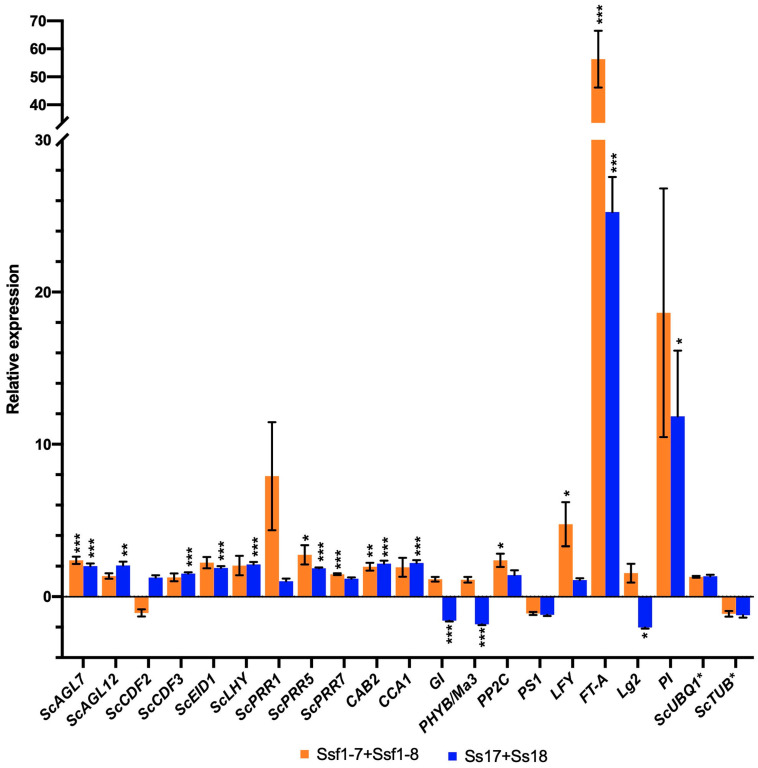
Relative expression of flowering related genes at the beginning of smut symptom and initiating floral structures after inoculation of smut fungi from plants materials included leaves and stalks. * Reference Genes. *p* < 0.001 (***), *p* < 0.01 (**) and *p* < 0.05 (*).

The results showed that *ScAGL7*, *ScPRR5*, *ScPRR7*, *CAB2*, *PP2C*, *LFY* and *FT-A* were significantly (*p* < 0.05) up-regulated in plants infected by Ssf1-7 + Ssf1-8 (Figure 6). In contrast, *ScAGL7*, *ScAGL12*, *ScCDF3*, *ScEID1*, *ScLHY*, *ScPRR5*, *CAB2*, *CCA1*, *GI*, *PHYB/Ma3*, *FT-A*, *Lg2* and *PI* genes were differentially expressed in Ss17 + Ss18-infected plants, with a statistically significant difference (*p* < 0.05) (Figure 6). Four genes (*ScAGL7*, *ScPRR5*, *CAB2* and *FT-A*) were commonly detected in infected plants, with significantly different (*p* < 0.05) expression patterns, as compared to the controls (Figure 6). This result confirmed that the smut fungi Ssf1-7 and Ssf1-8 can cause a plant host to enhance expression of flowering-related genes, likely contributing to the initiation of floral structures.

## 3. Materials and Methods

### 3.1. Isolation of the Causal Agent of Smut from Inflorescence of Sugarcane

One diseased sample with the symptom of smut-infected inflorescence was collected from sugarcane clone (no commercial variety) in a sugarcane experimental field, Sugarcane Research Institute, Guangxi Academy of Agricultural Sciences, Nanning, China (22.49° N, 108.18° E) in October 2018. Isolation of dikaryon colonies and mating-type haploid strains of the causal agent of smut was performed from the collected sample, using the method described by Yan et al. [26]. Growth conditions and media for isolates were followed by Yan et al. [26]. Growth curves of haploid strains and teliospore germination of *S. scitamineum* were followed with the method described by Zhong et al. [27]. The reported *S. scitamineum* sporidial strains, such as Ss17(*MAT-1*) and Ss18 (*MAT-2*) [26], served as controls in the pathogenicity assays.

### 3.2. Microscopic Observations

For microscopic observation, samples of floral structure with smut were detected and imaged by stereoscopic microscope (Nikon SMZ18, Tokyo, Japan). The isolated strains were placed on the glass slides, observed and imaged using an Axioimager Z1 microscope equipped with an AxiocamMRm camera (Carl Zeiss, Jenna, Germany), and sporidia shape, color and a total of 100 sporidia sizes for each isolated strain were analyzed.

### 3.3. Scanning Electron Microscope (SEM) Examination

Scanning electron microscopy (SEM) examination was conducted to understand the causal agent of sugarcane inflorescence. The floral structures with smut symptoms were mixed in FAA solution [formaldehyde (70%), glacial acetic acid and ethanol (5:5:90)]. The samples were dehydrated in an ethanol series, and the following steps were done as described by Marques et al. [28].

### 3.4. Pathogenicity Test

Six sugarcane commercial varieties with different resistance levels for smut infection, including GT42, GT44, GT49, GT55, LC05-136 and ROC22, were used to test the virulence of the smut pathogen isolates. GT44 and GT55 varieties belong to resistance in rating for smut infection, whereas GT42, GT49, LC05-136 and ROC22 were highly susceptible to smut and expected to show disease symptoms; among them, GT42 and LC05-136 are the main varieties in Guangxi. To save the preparatory work, shorten the period of the pathogenicity test and improve the efficiency of the pathogenicity test of smut fungus, disease-free tissue-cultured seedlings of the six sugarcane varieties with the height of 20–30 cm were used for the inoculation of the smut pathogen.

The pathogenicity test was conducted in a greenhouse, according to Yan et al. [26] with minor modifications. Two different mating type strains of the isolates were put into 50 mL of YePS liquid medium, incubated at 28 °C and shaken at 200 rpm (2 days). The cells of 2 different mating type strains were washed twice with distilled water, centrifuging at 4000 rpm for 5 min and then re-suspending in 20 mL of distilled water. One mL of a mixture of opposite mating type strains was inoculated into the disease-free tissue-cultured seedlings, with inoculation of the mixture of Ss17 and Ss18 as the positive control, which caused characteristic symptoms of sugarcane smut from the top of the plant cane [26] and water inoculation as the negative control. For each variety, 10 replicates were set for the smut fungus of floral infection, positive and negative controls. Re-isolation from infected floral structures were carried out according to Koch’s postulates.

### 3.5. Molecular Identification

Total DNA isolation of the smut pathogen isolates in sugarcane inflorescence was conducted by Yan et al. [26]. Primer pairs of ITS4 (TCCTCCGCTTATTGATATGC) and ITS5(GGAAGTAAAAGTCGTAACAAGG) were used to amplify the rDNA internal transcribed spacer (ITS) region of the isolates by PCR. The PCR-amplified products were analyzed by electrophoresis in 1.0% (*w*/*v*) of agarose gel. The amplified fragments were sequenced by Invitrogen Trading Co., Ltd., Shanghai, China. The nucleotide sequences were blasted and analyzed in NCBI (https://blast.ncbi.nlm.nih.gov/Blast.cgi, accessed on 1 January 2020). The pathogen was determined, finally, based on the identity of the sequence and the morphology of the isolates.

### 3.6. Flowering Induction of Sugarcane Variety LC05-136 with Smut Fungus Isolates

Sugarcane floral induction assay inoculated the smut isolates to disease-free tissue-cultured seedlings of sugarcane variety LC05-136 with the height of 20–30 cm, under the optimum temperature (20–35 °C) and relative humidity (70–80%) in the greenhouse for 6 months. Sugarcane floral induction assay was carried out twice in a year, from February to July and July to December, due to the intermediate day length plants. The inoculation method was described as above, and 20 replicates were performed. The reported *S. scitamineum* sporidial strains Ss17 and Ss18 [26] were mixed. They inoculated the seedlings, serving as the positive control for causing the characteristic symptoms of sugarcane smut from the top of the plant stalk. Water inoculation served as the negative control. Ten replicates each were performed for positive and negative controls.

### 3.7. Quantitative Real-Time PCR (RT-qPCR) Assessment of Flowering-Related Genes

Nineteen sugarcane homologous genes involved in photoperiod response and flowering time were selected, depending on the previous studies, and the relative expression levels of genes were analyzed under smut infection using qRT-PCR. Flowering-related genes, reference genes and their oligonucleotide primer pairs are presented in Table 1 [21,22,23]. Plants (LC05-136), including leaves and stalks, were harvested at the period of the beginning of regular smut symptoms (black whip) and initiating floral structures (Supplementary Figure S1**),** after inoculation of smut fungi for RNA extraction using the HiPure HP Plant RNA Mini Kit (Magentec, R4165-02, Guangzhou, China). The cDNA synthesis was performed with HiScript^®^ II Q RT SuperMix for qPCR (+gDNA wiper) (Vazyme Biotech Co., R223-01, Nanjing, China). The RT-qPCR assays were conducted using the CFX96 real-time PCR detection system (Bio-Rad, Hercules, CA, USA). The reaction was performed in a final volume of 20 µL, containing 10 µL of Universal SYBR qPCR Master Mix (Vazyme Biotech Co., Q711-02/03, Nanjing, China), 1 µL of cDNA and 0.4µL of primer pairs (10 μM). Amplification conditions of RT-qPCR were 3 min at 95 °C, followed by 45 cycles of 95 °C for 15 s and 72 °C for 20 s, followed by a melting curve from 55 °C to 60 °C. For the RT-qPCR of the 19 target genes, the 3 bio-replicates sampled in each smut infection treatment were performed with 3 technical replicates. The data analysis was performed as described by Manechini et al. [22].

## 4. Discussion

Sugarcane smut is one of the most important sugarcane diseases globally, and it can result in severe yield losses in sugarcane production and reduction in sugar content [29]. The most recognizable characteristic symptom of sugarcane smut is the emergence of a long, black whip from the top of the plant stalk. The whip-like structures can be from 10 mm to over 1 m in length and are composed of a central core of plant tissue surrounded by a layer of fungal tissue that develops black teliospores. Many tillering with spindly and erect shoots and small narrow leaves were produced in the affected plants [8]. This study observed a combination of floral structure and black whip in sugarcane plants. A curved, short, small and black whip-like structure emerged from the glumes of a single floret. The floral whips ranged from 3 to 20 mm in length and consisted of a layer of fungal tissue that forms black teliospores, similar to the observation by Sundar et al. [8] and Glassop et al. [23], and this was the first report in China. Plant floral development was interfered by other smut fungi. For example, smut fungus *U. maydis* affects vegetative and reproductive progress of maize by developing tumors on inflorescences and stems and leaves [30]. Tumor and phyllody development caused by *S.reilianum* result from changes in the floral developmental program in maize [31]. Hermaphroditism (i.e., development of pistils) in florets of male buffalo-grass was induced by the biotrophic pistil smut fungus *Salmacisia buchloeana* [32]. Other symptoms of sugarcane smut with leaf and stem galls and bud proliferation were reported by Sundar et al. [8]. The various symptoms of sugarcane smut disease may depend on the environmental conditions and/or the susceptibility/resistance of different sugarcane varieties to the fungal races.

In this study, we compared the teliospores and haploid sporidia morphology characteristics between the isolates of smut pathogen from the flower and the established *S. scitamineum* sporidial strains Ss17 and Ss18 [26], which usually cause smut on the top of sugarcane stalks. We found no noticeable difference in shape, color or sizes of teliospores and haploid sporidia between the flower isolates and the regular whip isolates Ss17 and Ss18. However, the smut strains Ssf1-7 and Ssf1-8 displayed stronger sexual mating ability than the reported strains Ss17 and Ss18, based on the mating assay. Furthermore, we further showed that newly isolated *S. scitamineum* sporidial strains Ssf1-7 and Ssf1-8 could induce sugarcane (LC05-136) flowering without teliospores/black whip formation and result in abnormal morphological modifications in the infected varieties of GT42, ROC22, GT44 and GT55, with less or no teliospores formation (Figure 3D–F,I). The mechanism of these interesting observations is worth further study, especially its stronger mating ability and how the fungus proliferates, without or with less teliospores formation.

Investigation of genetic diversity, including variations in virulence in different smut fungus populations, is especially important for breeding smut-resistant varieties and developing disease control strategies. Several studies suggested that the molecular variation in *S. scitamineum* was related to geographical origin [11,12]. In addition, three pathogenic races of *S. scitamineum* derived from different geographical groups in mainland China developed highly significant differences in pathogenicity [19,20]. On the contrary, although the different races of *S. scitamineum* have been described in some sugarcane planting countries [13,14,15,16,17,18], systematic investigation is still lacking on *S. scitamineum* distribution in different regions affected by environmental factors and/or sugarcane varieties. In this study, the newly identified *S. scitamineum* race displayed apparent differentiation of pathogenicity, compared to our previous isolated strains [26]. Furthermore, smut isolates collected from variety LC05-136 only developed black whips from the top of plant stalks post inoculation to sugarcane varieties, including LC05-136 (data not shown). Plants of variety LC05-136 can also be induced to form black whips from the top of plant stalks, but not induce flowers after infection by the mixture of Ssf1-7 + Ss18 or Ssf1-8 + Ss17 (data not showed). We speculate that the flowering induction ability of the fungus is determined by the interaction between the specific plant host and pathogen race, rather than by environmental factors, indicating a host-pathogen interaction; yet, we have minimal understanding at present.

Sugarcane is the intermediate day-length plant that can be induced to flower under decreasing day lengths [21,22]. In the present study, 80% of the Ssf1-7 + Ssf1-8 infected plants of LC05-136 formed flowers in 6 months, from July to December (day length decreases), whereas 30% of the infected LC05-136 induced flowering in the period of February to July (day length increases). We speculate that sugarcane can be regulated to perceive photoperiod changes by smut fungus of Ssf1-7 + Ssf1-8 stress, which causes sugarcane to switch from vegetative to reproductive flowering induction. The results of RT-qPCR further confirmed that the expression levels of six genes involved in the flowering in plants infected by smut fungus Ssf1-7 + Ssf1-8 were higher at the beginning of flowering, compared to uninfected plants. Maybe more flowering-related genes with high expression are detected in flower formation than initiating floral structures in infected plants post inoculation of smut fungus Ssf1-7 + Ssf1-8. Interestingly, for inoculation of the reported strains Ss17 + Ss18, 13 flowering-related genes with statistically significant difference were expressed in infected plants, compared to uninfected plants, through RT-qPCR (Figure 6). The finding is similar to the results demonstrated by Glassop et al. [23]. The expression level for six flowering related genes was increased in smut-infected plants, compared to the controls, but only in the sugarcane variety, i.e., Q174. Whereas, based on transcriptomic data, flowering-related genes, such as MADS-box TFs and FLOWERING LOCUS T (FT), are highly expressed in infected sugarcane plants after whip emission [33]. In *Arabidopsis thaliana*, FT encodes a small peptide that is regarded as the main constituent of florigen and promotes switching from vegetative to reproductive growth [34]. However, it remains a question why the plants’ variety of LC05-136 cannot be induced to flower by infection of the reported strains Ss17 + Ss18, but can be induced by inoculation of smut fungus Ssf1-7 + Ssf1-8, even if more flowering-related genes’ high expression was detected than that of sugarcane infected by smut fungus Ssf1-7 + Ssf1-8. We speculated that the reason is that there may be interaction between the specific plant host and pathogen race. In addition, sugarcane flowering is a complex physiological process affected by environmental variables, including latitude, altitude, photoperiod, temperature, moisture, nutrition, etc. [23,24,25,35]. Transcriptomic analysis revealed 76 sugarcane putative orthologs to flowering genes involved in the induction of sugarcane flowering, and 21 transcripts were differentially expressed under controlled photoperiodic conditions [22]. Furthermore, some flowering-related genes were reported displaying significant changes in expression within 24 h, and this may be also one of reasons, resulting in more flowering-related genes with high expression detected in smut fungus Ss17 + Ss18 infected sugarcane than that of smut fungus Ssf1-7 + Ssf1-8.

The sugarcane variety of LC05-136 has become one of the major sugarcane varieties in Guangxi. Planting areas accounted for more than 35% of the total, and sugarcane cultivar LC05-136 was not yet reported to flower under natural conditions in Guangxi, China. Moreover, the mechanisms of sugarcane flowering remain unclear, particularly sugarcane flowering induction by *S. scitamineum*. Therefore the smut fungus strains Ssf1-7 and Ssf1-8 in this study are of great research value and have a good application prospect in breeding and inducing flowering. The transcriptomic analysis of sugarcane variety of LC05-136 under the stress of smut fungus of *S. scitamineum*, pollen and stigma formation activity and unusual symptoms in other varieties will be interesting in our future investigations.

## 5. Conclusions

The causal pathogen of smut disease in sugarcane flower was identified as *S. scitamineum* in Guangxi, China, and its flowering-inducing ability was specific in sugarcane variety LC05-136. This sugarcane variety could be used for further investigation on the mechanisms of sugarcane flowering, genetic diversity of smut pathogen races and fungal–plant interactions.

## Figures and Tables

**Table 1:** Sugarcane putative flowering-related genes selected for gene expression analysis by RT-qPCR using sugarcane tissues collected at the beginning of smut symptom and flowering stage.

Gene	Gene Name	Forward Primer Reverse Primer	Forward Primer Reverse Primer
ScAGL7	AGAMOUS LIKE-7	GACGGTTCAGGCTCAGATT	GCTTTAATGAACGCACACCTC
ScAGL12	AGAMOUS LIKE-12	GAGATGGGCTATTCCTTCTGAC	CTCCTGAAGGGCTATGGTTTAT
ScCDF2	CYCLING DOF FACTOR 2	CTGTGATGGTGCCAGGTAAA	GCACAAGTGGGTATGGAAATG
ScCDF3	CYCLING DOF FACTOR 3	TCAGGTTTCGACTGGAATGG	AAGGAGATGAGAAGGCAGAAAG
ScEID1	EID1-like1	TTCTGAGGACACAAAGGAAGAG	CAAAGAGAAAGGCAGCTAGGA
ScLHY	LATE ELONGATED HYPOCOTYL	GTGTCTCTCCACACAGAGTTAAA	TTGTCCGCATCTACATCACTAC
ScPRR1	PSEUDO-RESPONSE REGULATOR 1	CTCAAGCACATACACCACCA	ATGCCGATGACCACACATT
ScPRR5	PSEUDO-RESPONSE REGULATOR 5	ACAGAAGCAGAAACTGACTCG	CCTTCAGTCTTACCAGTCCAAT
ScPRR7	PSEUDO-RESPONSE REGULATOR 7	CAGTGGCAGTGGAAGTGAAA	CATTGAGTCCGACACTGAAGTC
CAB2	CHLOROPHYLL a/b BINDING PROTEIN	TGATACATGCATCTGTGCTGCTT	TGGTAGGCCGGCGTGTAG
CCA1	CIRCADIAN CLOCK ASSOCIATED 1	ATGAGAAGGTGAAGCAAGCCT	TGCTTCTAAATCTGCGGTGGT
GI	GIGANTEA	ACATGCCGAAGGAGTTGAAG	GTGCAGTGGCATCGATAGTG
PHYB/Ma3	PHYTOCHROME B/MATURITY GENE 3	GCCTATATTTGCCAGGAGA	CTTGGACATCTGTTCCTCA
PP2C	PROTEIN PHOSPHATASE 2C	AGACAGCAGAGGTGGACATGAA	CGTCTTCTAGCCTCTGGAAACA
PS1	PHOTOSYSTEM 1	CCTGAAGGCCCCATCCAT	GGAGGGTCGTCTCCTTGTGA
LFY	Leafy	TCACCAGCTTCCTCGGTTTA	AACCCTCTTGCCAAATAGCC
FT-A	Flowering locus T (FT) homologue A	GACATGCGCACCTTCTACAC	CGAGCTGTTGGAAGAGCAG
Lg2	Liguleless 2	TTGCCAACTACACTGCTCTC	TCTGGTGCAACGTCTGCTG
PI	Pistillata	GCACAAGAGCCTTAGTGCAG	AGATCTTCACCTTTCAGGTGC
ScUBQ1 *	UBIQUITIN 1	AGCCTCAGACCAGATTCCAA	AATCGCTGTCGAACTACTTGC
ScTUB *	TUBULIN	CTCCACATTCATCGGCAACTC	TCCTCCTCTTCTTCCTCCTCG

* Reference genes.

## Data Availability

The data presented in this study are available within the article and its Supplementary Material.

## Supplementary Materials

The following supporting information can be downloaded at: https://www.mdpi.com/article/10.3390/plants12020316/s1, Table S1: The ITS DNA sequences of *S. scitamineum* strains; Table S2: The number of days for morphological modifications in cane plants infected by smut fungus *S. scitamineum* in July to December in 2020; Table S3: The number of days for morphological modifications in cane plants infected by smut fungus *S. scitamineum* in February to July in 2021; Figure S1: Plants were harvested at the beginning of smut symptoms(A) and initiating floral structures(B) after inoculation of smut fungi for RNA extraction; Figure S2: Grow curves of different mating type cells of *S. scitamineum*.
