# Supplementary material for: Variety-Specific Flowering of Sugarcane Induced by the Smut Fungus Sporisorium scitamineum
Source: Plants (Basel). 2023 Jan 9;12(2):316. doi: 10.3390/plants12020316 (PMC9863003; doi:10.3390/plants12020316)
Supplement: Supplementary file 1 [file plants-12-00316-s001.zip › plants-2056126-supplementary.pdf]

Table S1 The ITS DNA sequences of *S. scitamineum* strains.

Strains	GenBank accession number	Primer pairs	Sequences
Ssf1-7	MZ470432	ITS4/<br>ITS5	TGGAAGTAAAAGTCGTAACAAGGTATCTGTAGGTGAACCTGCAGATGGATCATTTCGAGTGAAACCTTTTTTCCGAGGTGTGGCTCG<br>CACCTGTCTAACTAAACTGGGCTACCTATTTCAACACGGTTGCATCGGTTGGGTCTGCCAAACAGTGCACGAAAGTACCTGTGGAGG<br>CAGCCCGATAATCTACCAAAACACTTTTGATGGTCTAGGATTTGAAAGTATTTAACATTTTACGACTGGTAATGCGGTCGTCTAAAATC<br>TAAAAAACAACTTTTGGCAACGGATCTCTTGGTTCTCCCATCGATGAAGAACGCAGCGAATTGCGATAAGTAATGTGAATTGCAGAA<br>GTGAATCATCGAATCTTTGAACGCACCTTGCGCTCCTTGCAGATCTAATCTGGGGAGCATGCCTATTTGAGGGCCGCGAATTGTTTCG<br>AACGCACGCTTTTTTATTACGAAAGAGCTGGCGGATCGGTAGTGAGGGTTTTGCCATTTACCGTGGCTCCCTCGAAATGCATTAGTGC<br>ATCCATTTGACAGGCAAAGACGGACGAAGGCTCGACTTTTGGCCCATCTTCCCTGCCAGGTTTTGATAATATCAGGACTTTGGTGGT<br>GAGGATGAGCAAGAAGCTGGACGCGACGGCCTTTGCTGATTGGAGTGCTTCTGAACACCGCCCTAGCTAATTTTTTATTTTATTTTTT<br>TGGCTAGGGAATAATTCAAATCGGCCTCAGATAGGTAGGACTACCCGCTGAACTTAAGCATATCAATAAGCGGAGGAA
Ssf1-8	MZ470431	ITS4/<br>ITS5	TGGAAGTAAAAGTCGTAACAAGGTATCTGTAGGTGAACCTGCAGATGGATCATTTCGAGTGAAACCTTTTTTCCGAGGTGTGGCTCG<br>CACCTGTCTAACTAAACTGGGCTACCTATTTCAACACGGTTGCATCGGTTGGGTCTGCCAAACAGTGCACGAAAGTACCTGTGGAGG<br>CAGCCCGATAATCTACCAAAACACTTTTGATGGTCTAGGATTTGAAAGTATTTAACATTTTACGACTGGTAATGCGGTCGTCTAAAATC<br>TAAAAAACAACTTTTGGCAACGGATCTCTTGGTTCTCCCATCGATGAAGAACGCAGCGAATTGCGATAAGTAATGTGAATTGCAGAA<br>GTGAATCATCGAATCTTTGAACGCACCTTGCGCTCCTTGCAGATCTAATCTGGGGAGCATGCCTATTTGAGGGCCGCGAATTGTTTCG<br>AACGCACGCTTTTTTATTACGAAAGAGCTGGCGGATCGGTAG<br>TGAGGGTTTTGCCATTTACCGTGGCTCCCTCGAAATGCATTAGTGCATCCATTTGACAGGCAAAGACGGACGAAGGCTCGACTTTTG<br>GCCCATCTTCCCTGCCAGGTTTTGATAATATCAGGACTTTGGTGGTGAGGATGAGCAAGAAGCTGGACGCGACGGCCTTTGCTGATT<br>GGAGTGCTTCTGAACACCGCCCTAGCTAATTTTTTATTTTATTTTTTTGGCTAGGGAATAATTCAAATCGGCCTCAGATAGGTAGGACT<br>ACCCGCTGAACTTAAGCATATCAATAAGCGGAGGAA
Ss17	MZ470434	ITS4/<br>ITS5	TGGAAGTAAAAGTCGTAACAAGGTATCTGTAGGTGAACCTGCAGATGGATCATTTCGAGTGAAACCTTTTTTCCGAGGTGTGGCTCG<br>CACCTGTCTAACTAAACTGGGCTACCTATTTCAACACGGTTGCATCGGTTGGGTCTGCCAAACAGTGCACGAAAGTACCTGTGGAGG<br>CAGCCCGATAATCTACCAAAACACTTTTGATGGTCTAGGATTTGAAAGTATTTAACATTTTACGACTGGTAATGCGGTCGTCTAAAATC<br>TAAAAAACAACTTTTGGCAACGGATCTCTTGGTTCTCCCATCGATGAAGAACGCAGCGAATTGCGATAAGTAATGTGAATTGCAGAA<br>GTGAATCATCGAATCTTTGAACGCACCTTGCGCTCCTTGCAGATCTAATCTGGGGAGCATGCCTATTTGAGGGCCGCGAATTGTTTCG<br>AACGCACGCTTTTTTATTACGAAAGAGCTGGCGGATCGGTAGTGAGGGTTTTGCCATTTACCGTGGCTCCCTCGAAATGCATTAGTGC<br>ATCCATTTGACAGGCAAAGACGGACGAAGGCTCGACTTTTGGCCCATCTTCCCTGCCAGGTTTTGATAATATCAGGACTTTGGTGGT<br>GAGGATGAGCAAGAAGCTGGACGCGACGGCCTTTGCTGATTGGAGTGCTTCTGAACACCGCCCTAGCTAATTTTTTATTTTATTTTTT<br>TGGCTAGGGAATAATTCAAATCGGCCTCAGATAGGTAGGACTACCCGCTGAACTTAAGCATATCAATAAGCGGAGGAA
Ss18	MZ470433	ITS4/<br>ITS5	TGGAAGTAAAAGTCGTAACAAGGTATCTGTAGGTGAACCTGCAGATGGATCATTTCGAGTGAAACCTTTTTTCCGAGGTGTGGCTCG<br>CACCTGTCTAACTAAACTGGGCTACCTATTTCAACACGGTTGCATCGGTTGGGTCTGCCAAACAGTGCACGAAAGTACCTGTGGAGG<br>CAGCCCGATAATCTACCAAAACACTTTTGATGATCTAGGATTTGAAAGTATTTAACATTTTACGACTGGTAATGCGGTCGTCTAAAATC<br>TAAAAAACAACTTTTGGCAACGGATCTCTTGGTTCTCCCATCGATGAAGAACGCAGCGAATTGCGATAAGTAATGTGAATTGCAGAA<br>GTGAATCATCGAATCTTTGAACGCACCTTGCGCTCCTTGCAGATCTAATCTGGGGAGCATGCCTATTTGAGGGCCGCGAATTGTTTCG<br>AACGCATGCTTTTTTTATTACGAAAGAGCTGGCGGATCGGTAGTGAGGGTTTTGCCATTTACCGTGGCTCCCTCGAAATGCATTAGTG<br>CATCCATTTGACAGGCAAAGACGGACGAAGGCTCGACTTTTGGCCCATCTTCCCTGCCAGGTTTTGATAATATCAGGACTTTGGTGGT<br>GAGGATGAGCAAGAAGCTGGACGCGACGGCCTTTGCTGATTGGAGTGCTTCTGAACACCGCCCTAGCTAATTTTTTATTTTATTTTTT<br>TGGCTAGGGAATAATTCAAATCGGCCTCAGATAGGTAGGACTACCCGCTGAACTTAAGCATATCAATAAGCGGAGGAA

Table S2 The number of days for morphological modifications in cane plants infected by smut fungus *S. scitamineum* in July to December in 2020

Fungus	No.	Duration of pre-symptom stage(day)	Type of symptom
Ssf1-7+Ssf1-8	1	65	flowering and smut free
	2	69	flowering and smut free
	3	71	flowering and smut free
	4	72	flowering and smut free
	5	74	flowering and smut free
	6	75	flowering and smut free
	7	77	flowering and smut free
	8	78	flowering and smut-free
	9	81	flowering and smut free
	10	81	flowering and smut free
	11	84	flowering and smut free
	12	89	flowering and smut free
	13	96	malformed spindle
	14	106	flowering and smut free
	15	113	malformed spindle with smut
	16	115	combination of floral structures and smut
	17	119	flowering and smut free

	18	136	combination of floral structures and smut
	19	-	no symptom
	20	-	no symptom
Ss17+Ss18	1	69	culmicolous smut
	2	72	culmicolous smut
	3	72	culmicolous smut
	4	73	culmicolous smut
	5	80	culmicolous smut
	6	92	culmicolous smut
	7	98	culmicolous smut
	8	118	culmicolous smut
	9	-	-
	10	-	-
Negative control	-	-	-

Table S3 The number of days for morphological modifications in cane plants infected by smut fungus *S. scitamineum* in February to July in 2021

Fungus	No.	Duration of pre-symptom stage(day)	Type of symptom
Ssf1-7+Ssf1-8			
	1	110	flowering and smut free

	2	115	flowering and smut free
	3	118	flowering and smut free
	4	120	flowering and smut free
	5	121	flowering and smut free
	6	124	combination of floral structures and smut
	7-20	-	no symptom
Ss17+Ss18	1	75	culmicolous smut
	2	88	culmicolous smut
	3	96	culmicolous smut
	4	99	culmicolous smut
	5	109	culmicolous smut
	6	112	culmicolous smut
	7-10	-	-
Negative control	-	-	-

Figure Legends

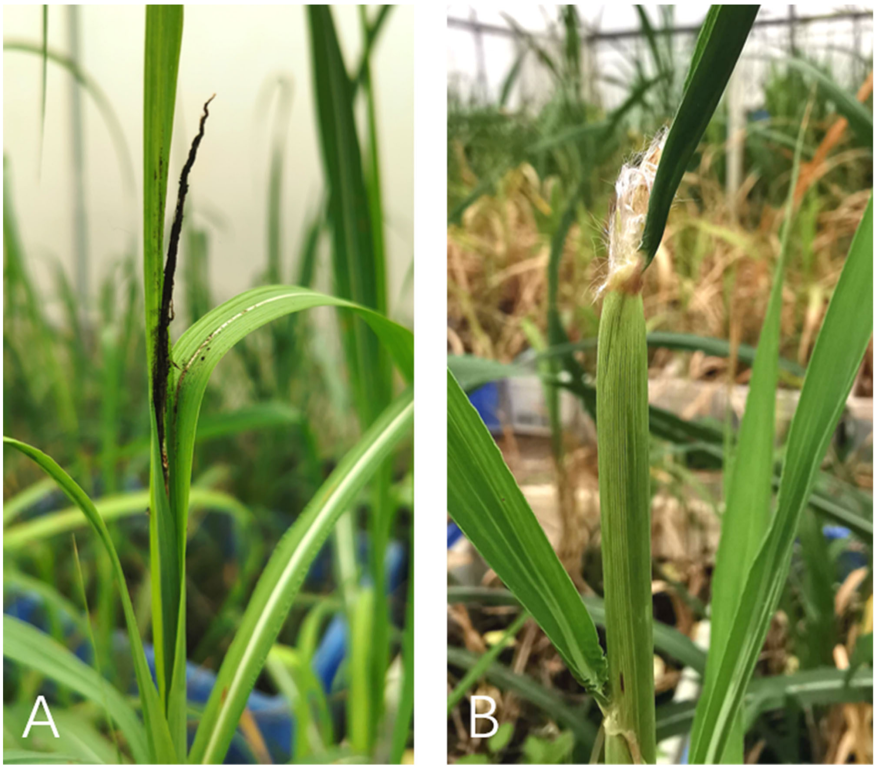

Figure S1 Plants were harvested at the beginning of smut symptoms(A) and initiating floral structures(B) after inoculation of smut fungi for RNA extraction

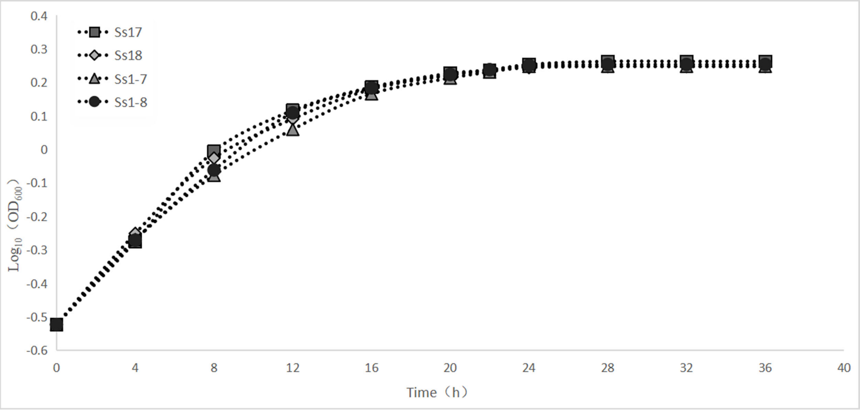

Figure S2 Grow curves of different mating type cells of *S.scitamineum*
